# Aural foreign body extraction in children: a double-edged sword

**DOI:** 10.11604/pamj.2015.20.186.5218

**Published:** 2015-02-27

**Authors:** Oyebanji Olajuyin, Oladele Simeon Olatunya

**Affiliations:** 1 Department of Ear, Nose and Throat Ekiti State University Teaching Hospital, Ado-Ekiti, Nigeria; 2 Department of Paediatrics, Ekiti State University Teaching Hospital, Ad-Ekiti, Nigeria

**Keywords:** Aural foreign body, children, complications, hearing loss

## Abstract

**Introduction:**

Foreign body insertion into the ear in children is common world-wide. The goal of this work is to describe the procedural complications of aural foreign body extraction in children.

**Methods:**

A retrospective analysis of records of children with aural foreign bodies was conducted. Patients’ bio data, type of foreign bodies, referrals, techniques of removal and complications were extracted from the case files. The foreign bodies were categorized into graspable and non-graspable objects. Patients with complications caused directly by the foreign body were excluded.

**Results:**

There were 136 cases. Eighty-seven (64.0%) were males while forty-nine (36.0%) were females. Their age range from 5 days to 16 years with 109 (80.2%) aged below 8 years. Eighty-nine (65.4%) and 47 (34.6%) cases were treated by otolaryngologists and non-otolaryngologists with a complication rate of 15.7% and 68.1% respectively. One case suffered severe hearing loss following complicated attempt at removing foreign body in the only hearing ear. Overall, the complication rate was higher (44.4%) with removal of non-graspable than (28.6%) with graspable objects.

**Conclusion:**

Procedural complication is an ever-present hazard of aural foreign body extraction in children. Its occurrence can be prevented or largely reduced if health care-givers know their limitation based on their clinical skills and acquaint themselves with established criteria for referral. As a rule, we suggest that, foreign body in the only hearing ear and failed attempted first removal should be considered criteria for otolaryngologic referral.

## Introduction

Foreign body insertion into the ear in children is common world-wide. Children being curious and experimental in their activities, tend to insert foreign bodies into own and/or each other's ears often without the knowledge of parents or guardians. Safeguarding their ears against foreign body insertion therefore becomes a herculean task. Among the objects found culpable are beads, buttons, plastic toys, pebbles, popcorn kernels, paper, eraser, and vegetable materials [1–3]. Insects are more common in patients older than 10 years [4]. Except there is immediate eye witness account, diagnosis is often delayed as the victim often don't present early. Aural foreign body in children is often an incidental finding [4]. Others may present with ear pain, discharge, bleeding, hearing loss or tinnitus [4, 5]. The goal of removal is to preserve the integrity of the ear while the foreign body is being removed. Removal may be done with or without general anaesthesia. A variety of instrumentation should be available for extraction given the variety of objects encountered. These include forceps, cerumen loop, right-angled ball hook and Frazier tip suctions [1]. The use of microscope is an added advantage. Some aural foreign bodies can be removed by irrigation. In every case, bright illumination and patient's immobilization are essential for successful outcome. The first attempt at removal is critical because success rates markedly decrease after the first failed attempt [3]. And failed attempted removal results in higher complication rate [6]. These could be canal abrasion, laceration, bleeding, perforation of tympanic membrane, ossicular chain destruction and hearing loss [2, 7]. In addition to instrumentation, type, shape and location of the foreign body, complications appear to be related to the level of clinical skill of individual health-care givers. As noted by workers, there is a significant difference in complication rate between patients treated by Otolaryngologists and Non-Otolaryngologists [7, 8]. Thus, non-otolaryngologists must be aware of their limitations and refer to specialists as appropriate. The criteria for Otolaryngologic referral are well outlined by Ansley and Cunningham [1]. In the developing countries however, poor referral system, dearth of skilled health care workers and lack of appropriate instruments contribute significantly to the frequency and fatality of complications associated with aural foreign body extraction. Thus, in this study, we describe the procedural complications of aural foreign bodies to sensitize the health care givers on the scope and magnitude of these complications with a view to prevent or reduce its incidence.

## Methods

Study setting: This study was conducted at Ekiti State University Teaching Hospital, a tertiary referral hospital with Otolaryngological and Paediatric care services. The hospital provides specialist care for the host community and neighbouring States.

Study Design: A retrospective analysis of records of children with aural foreign bodies was conducted between January 2011 and June 2014. All consecutive cases whose records contained the relevant clinical data were recruited into the study. The information which included Age, Sex, type of foreign body, referrals, failed attempted removal, technique of removal and complications were extracted from the case files. The foreign bodies were categorized into graspable and non-graspable objects.

Exclusion criteria: Excluded were cases with complications found to have resulted from the presence of the foreign body in the ear (non-procedural complications).

Ethical Consideration: The study was approved by the Ethics and Research committee of the Ekiti State University Teaching Hospital.

Data Analysis: The data generated was entered into personal computer and simple descriptive statistics was performed using SPSS Version 14.

## Results

**Figure 1:**
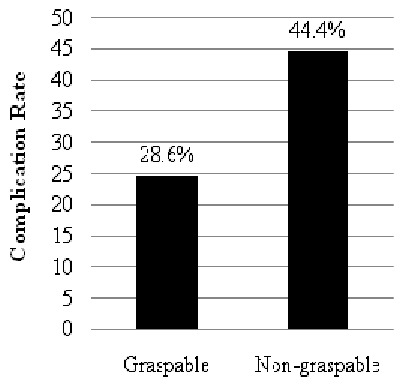
Complication Rate in relation to graspability of the objects

**Figure 2:**
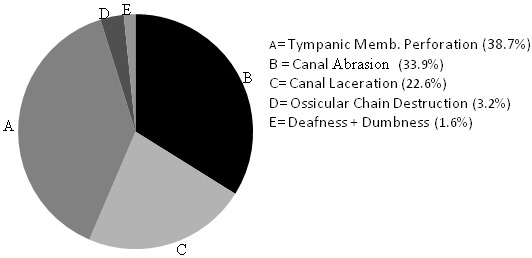
Distribution of the various types of complications

A total number of 136 cases with relevant clinical data were analyzed. Eighty-seven (64.0%) were males while forty-nine (36.0%) were females. Their age range from 5 days to 16 years with 109 (80.2%) aged below 8 years. All the cases were unilateral insertions (right more than the left) and no multiple insertions. Eighty-nine (65.4%) cases consisting of 62 graspable and 27 non-graspable were treated primarily by otolaryngologist. Of this number, 14 (15.7%) had a total of 16 complications: 7 canal abrasions, 6 canal laceration, 3 perforated tympanic membrane. All the removals were done using customized otolaryngological instruments. The remaining 47 (34.6%) had attempted removal done by non-otolaryngologists. This comprises of 36 (26.5%) attempted by other health care workers and 11 (8.1%) by parents/guardians. Attempted removal by parents and guardians were done with non-medical instruments such as broomstick, matchstick, twig and hairpin. As shown in Table 1, 32 (68.1%) of the 47 cases (29 graspable, 18 non-graspable), who had attempted removals done by non-otolaryngologists (other health care workers and parents/guardians) had a total of 46 complications: 14 canal abrasions, 8 canal laceration, 21 perforated tympanic membrane, 2 missing ossicles and one case of severe hearing loss following complicated attempt at removing foreign body in the only hearing ear by a general practitioner at a lower health care level. The hearing loss over a period of time culminated in the loss of speech leaving the child deaf and dumb. Thirty-four (25%) of all the cases required general anaesthesia for removal with about 56% of them less than 7years of age. Overall, there were 91 graspable and 45 non-graspable foreign bodies. Of the graspable foreign bodies, 26 (28.6%) had complications whereas 20 (44.4%) of the non-graspable objects were associated with complications (Figure 1). The most frequent complication is tympanic membrane perforation accounting for 38.7% (Figure 2) of which 54% were associated with non-graspable objects.

**Table 1:** Complication rate among cases by management groups

Cases and Complications (N = 136/46)	Otolaryngologists	Non-otolaryngologists	Total N = 136 (n%)
Number of cases managed	89	47	136 (100%)
Number of cases with complications	14	32	46 (33.8%)
Complication rate	15.73%	68.09%	
**Types of Complications**:			**Total N = 62 (n%)**
Perforated Tympanic Membrane	3	21	24 (38.7%)
Canal Abrasions	7	14	21 (33.9%)
Canal Laceration	6	8	14 (22.6%)
Missing Ossicles	0	2	2 (3.2)
Severe hearing loss and Dumbness	0	1	1 (1.6%)

**Note:** some patients had more than one type of complications

## Discussion

Foreign body insertion into the ear in children is increasingly becoming common. This could be attributed partly to the availability of diverse, handy, attractive and miniature items in modern day society. The accessibility to modern day communication devices and ammunitions has equally changed the epidemiological outlook of aural foreign bodies among children. Al-Juboori reported seven cases of Bluetooth devices that got stuck in the ears of students who inserted the device into their ears to cheat during examinations by receiving answers through the device with accomplice outside the examination hall [9]. Also, reported is a bullet inserted into the ear by a 6-year-old child [10]. Although, such bizarre foreign bodies were not found in our own study, their occurrences and complications could be major otological problems to grapple with in future. In the present study, 136 cases of aural foreign bodies were analysed. Of this, 89 (65.4%) were treated primarily by otolaryngologist while 47 (34.6%) had earlier been attempted by non-otolaryngologits. Analysis shows that 32 (68.1%) of the 47 cases attempted by non-otolaryngologists had complications whereas only 14 (15.7%) of the 89 cases treated by otolaryngologists were associated with complications. This is consistent with the result of other workers that removals by non-otolaryngologists are associated with higher complication rate than those of otolaryngologists [7, 8]. As noted by Fasunla et al, the level of clinical skill appears to be a major factor in the different complication rate recorded in the two groups [8]. Furthermore, the availability of a variety of otological instruments including operating microscope appears to contribute significantly to the lower complication rate among otolaryngologists. The increased usage of otomicroscope and successful removal of aural foreign bodies by otolaryngologists had earlier been reported by workers [11, 12]. Thus, failure to use specialised instruments and subsequent difficulty of discerning the complexity of certain foreign bodies in the ear might have accounted for the high complication rate among non-otolaryngologists.

In this study, the complication rate with removals of non-graspable objects was higher (44.4%) than removals of graspable objects (28.6%). This is in keeping with previous studies [13, 14]. In the cases of Scott and Richard, the success rate was significantly lower for firm, rounded items [14]. Apart from being difficult to grasp, the firm, smooth, non-graspable foreign bodies are more likely to slip deep in close contact with the tympanic membrane hence the lower success and greater complications rates associated with this type of foreign bodies. An impact analysis also demonstrated in this study that the most severe complications were associated with the non-graspable foreign bodies. As found, 54% of the tympanic membrane perforations were associated with non-graspable foreign bodies. Also, the two missing ossicles and the severe hearing loss recorded were associated with removal of non-graspable bodies. This highlights the need to always appraise the nature and location of aural foreign bodies in the external auditory canal before removal. It also warns that only a technique deemed to be safe and most effective should be used for removal of such foreign body. As a treatment option, non-graspable foreign body can safely be removed by irrigation if the tympanic membrane is intact provided the foreign body is free in the canal, non-hygroscopic and non-electrolytic. Also, coating the foreign body with drops of hydrophobic lubricant such as olive oil has been found by the authors to facilitate removal by irrigation. It is of interest to note that parents and guardian in-spite of their lack of skill and appropriate equipments, also poked blindly into the ears to extract foreign bodies. Apart from being unskilled, the use of objects such as broomstick, twig or matchstick to extract foreign bodies by this group of care-givers may inadvertently push and wedge the foreign body deeper in the external auditory canal. This will invariably convert what hitherto could have been a simple into a more difficult procedure thereby increasing the risks of procedural complications. It is equally worrisome to find two cases of missing ossicles following treatment by non-otolaryngologists. Okeowo in his book illustrated an incus removed by a general practitioner who mistook the middle ear bone for foreign body in the ear [15]. Of great concern however is the complete loss of hearing following complicated attempt at removing foreign body in the only hearing ear of an 8-year old girl by a general practitioner. The hearing loss over a period of time culminated in the loss of speech leaving the child deaf and dumb. The reason for such sudden hearing loss could be due to complication arising from the unskilled foreign body extraction or mere progression of a hitherto undiagnosed mild congenital hearing loss brought to the fore by the procedure. However, the timing of the recognition of the problem by the mother coinciding with the procedure in question leads much to be desired. Although, a fore-knowledge that the patient had only one hearing ear may not have precluded the otologic accident, preliminary diagnosis by simple hearing assessment would have guided the physician on safe removal or referral to otolaryngologist. Since such clinical entity may not be rare in clinical practice, routine performance of clinical test of hearing before removing foreign body in the ear may be worthwhile and if the foreign body is found to be in the only hearing ear, such case should be referred for otolaryngologic extraction.

As found in the current study, there was no significant difference in the rate of procedural complications between foreign bodies of less or greater than 24-hour duration. However, this observation is not limited to the current study as similar pattern had earlier been described by other researchers [16]. Thus, the risk of procedural complication is independent of the duration of insertion rather factors such as skill, instrumentation, and nature of the objects were its key determinant factors. Thirty-four (25%) of all the removals in this study were done under general anaesthesia. Age has been considered the most significant factor associated with the need for general anaesthesia [1]. In their study, Ansley and Cunningham noted that 30% of the patients underwent operative foreign body removal [1]. Of this number, 88% were less than 7 years of age. In our own study however, about 56% of those who required general anaesthesia were less than 7 years. Although, general anaesthesia reduces the risk of procedural complications in young children, it should be noted that age alone is not an absolute indication for general anaesthesia which in itself constitutes a risk. Where the object is graspable and in a position that allows safe removal in young children, such foreign body should be removed in the ambulatory setting. Whereas, foreign bodies in any age whose contour, composition or location in the canal predisposes to traumatic procedure should be removed under general anaesthesia. As a guide, the established criteria [1], are valuable for health care professional in choosing between operative or ambulatory removal. It is pertinent to note that about 80% of the victims in this study were on the verge of speech acquisition. Since hearing is a prerequisite for the acquisition of speech, injury to the auditory pathways will not only affect hearing but also ability to acquire speech. This and other known hazards are what make aural foreign body extraction in children a double-edged sword.

## Conclusion

Procedural complication is an ever-present hazard of aural foreign body extraction in children. Its occurrence can be prevented or largely reduced if health care-givers know their limitation based on their clinical skills and acquaint themselves with established criteria for referral. As a rule, foreign body in the only hearing ear and failed attempted first removal should be considered criteria for otolaryngologic referral. Also, we suggest that routine clinical tests of hearing should be performed in children with foreign body in the ear before removal.
